# Cannabis and Amphetamine Use Among Adolescents in Five Asian Countries

**DOI:** 10.5195/cajgh.2017.288

**Published:** 2017-12-07

**Authors:** Karl Peltzer, Supa Pengpid

**Affiliations:** 1 HIV/AIDS/STIs/and TB (HAST), Human Sciences Research Council, Pretoria, South Africa; 2 Department of Research and Innovation, University of Limpopo, Turfloop, South Africa; 3 ASEAN Institute for Health Development, Mahidol University, Salaya, Phutthamonthon, Nakhonpathom, Thailand

**Keywords:** Cannabis use, Amphetamine Use, Adolescents, Asia

## Abstract

**Introduction:**

There has been a global increase in illicit drug use among young people. The aim of this study was to estimate the prevalence of lifetime cannabis and amphetamine use, as well as to explore factors associated with substance use among adolescents in five Asian countries: Iraq, Kuwait, Malaysia, Mongolia, and Vietnam.

**Methods:**

38,941 school children (mean age 15.4 years, SD=1.5) completed the cross-sectional Global School-Based Student Health Survey (GSHS). Topics covered in the questionnaire included cannabis and amphetamine use. Personal, parental, and environmental attributes were explored as predictors of cannabis and amphetamine use. Logistic regression was used to assess the contribution of potential predictors on lifetime cannabis and lifetime amphetamine use.

**Results:**

Overall, the prevalence of lifetime cannabis use was 0.9% and lifetime amphetamine use was 1.0% among research participants. Cannabis use was influenced by male gender (Kuwait, Mongolia), parental smoking habits (Kuwait, Iraq), and current cigarette smoking in all countries. Amphetamine use was associated with suicidal ideation (Kuwait, Malaysia, Vietnam), school truancy (Malaysia, Mongolia, Vietnam), being a victim of physical assault (Kuwait, Mongolia), bullying victimization (Iraq, Malaysia, Vietnam), as well as anxiety and current cigarette use in all countries.

**Conclusions:**

Our preliminary results show the importance of personal attributes such as mental distress and environmental stressors on lifetime cannabis and lifetime amphetamine use. Future prospective studies are needed to identify causal relationships among personal attributes, parental attributes, environmental stressors, and illicit substance use.

Previous research suggested that substance use among youth under the age of 24 may have negative effects on cognitive and emotional development in the transition from adolescence to early adulthood.^1^ The initiation of substance use usually takes place during adolescence, mostly in the form of tobacco and alcohol use prior to the use of illicit drugs.^1^ A recent review reported that the global burden of disease attributable to substance use among adolescents and young adults has substantially increased.^1^ There are significant gaps in the literature regarding epidemiological data on the extent of illicit drug use among adolescents in Asia, which we are addressing in this paper.

The World Health Organization (WHO), in collaboration with the Centers of Disease Control and Prevention (CDC) and national governments, have been conducting Global School-based Health Survey (GSHS) in various Asian countries to generate epidemiological data on health behaviors, including illicit drug use.^2^ Since 2010, a new module on cannabis and amphetamine use was added to GSHS, which was implemented in Iraq and Kuwait in the Middle East, in Mongolia in Central Asia, and in Malaysia and Vietnam in Southeast Asia.^2^

Based on the WHO ATLAS on substance use disorders,^3^ the estimated 12-month prevalence of drug use disorders in 2004 was 0.24% among women (15 years and over) and 0.66% among men (15 years and over) in Iraq. For Mongolia, the prevalence was 0.15% among women and 0.61% among men, and for Vietnam it was 0.10% among women and 0.47% among men.^3^ Al-Hemiary et al.^4^ reported that there was an increase in illicit drug use, including cannabis (in the form of hashish) and methamphetamines in Iraq in the decade prior to 2012. In a study among male university students in Kuwait, the prevalence of lifetime illicit drug use was 14.4%.^5^ In previous national school surveys, lifetime cannabis use was 1.5% among males and 0.4% among females (aged 12–19 years) in Malaysia,^6^ and the prevalence of lifetime illicit drug use among adolescents (12–19 years) was 1.7% in Malaysia.^7^ In a local survey in Malaysia conducted in 1979, Spencer and Navaratnam^8^ found that 11% of secondary students (12–19 years) had prior experience of drug use (mostly cannabis). In Vietnam, amphetamine use continues to increase, especially among adolescents in major cities.^9^

In a previously published systematic review of cannabis use in high school and college students (≤18 years) in Iran, the prevalence of lifetime cannabis use was 5.0% (95% CI = 3.0%,7.0%).^10^ In a study focusing on high school students (mean age 15.0 years, SD=3.7) in Eastern India, the lifetime illicit drug use was 6.1% among rural and 0.6% among urban students.^11^ Lifetime illicit drug use was reported to be 7.9% among adolescents (15–18 years) attending primary health care centers in Saudi Arabia.^12^ In 38 European and North American countries, 19.9% of adolescents (22.9% of males and 17.0% of females) reported lifetime cannabis use.^13^ Among 15–16 year olds in Europe, lifetime amphetamine use ranged from 1% in Finland, Norway and Romania to 6% in Bulgaria and Latvia.^14^ Only a few studies were conducted in Asian countries, and information on the types of illicit drugs included in these studies was limited.^15^

Risk factors for cannabis and/or amphetamine use among adolescents can be grouped following an ecological framework^16^ into 1) sociodemographic factors, 2) personal attributes, 3) parental attributes, 4) environmental stressors, and 5) peer factors. Sociodemographic factors influencing drug use include male gender^17^,^18^ and age.^19^ Personal attributes may include mental distress,^20^–^22^ smoking,^7^,^18^ school truancy,^7^,^21^ and lack of peer support.^7^ Parental attributes may include parental substance use,^23^ lack of parental support, including lack of parental monitoring,^7^,^21^,^24^ and lack of parental connectedness.^21^ Environmental stressors may include hunger, lower economic status,^18^,^21^,^25^ bullying, fighting,^17^,^19^ and aggression.^26^

The aim of this study was to estimate the prevalence of cannabis and amphetamine use and explore factors associated with drug use among adolescents in five Asian countries. It was hypothesized that the prevalence of lifetime cannabis and amphetamine use was lower in Asian countries than in Europe and North America.

## Methods

### Participants

This study was a secondary analysis of preexisting data from the GSHS from five Asian countries (limited to countries that utilized the cannabis and amphetamine use module). The purpose of the GSHS is to provide nationally representative data on health behaviors and protective factors among students aged 13–17 years.^2^ The GSHS used a two-stage (schools and classrooms) cluster sampling design to produce nationally representative samples of students.^2^

Students were requested to complete a self-administered questionnaire under the supervision of trained survey administrators.^2^ National Ethics Committees (e.g., in Kuwait: The Ministry of Health; in Malaysia: the Medical Research and Ethics Committee, Ministry of Health Malaysia; in Mongolia: the Committee on Ethics, Ministry of Health) approved the study protocol, and written informed consent was obtained from the students, parents, and/or school officials.^2^

### Questionnaire

The GSHS questionnaire (translated into the national languages of the study countries) utilized in this study consisted of modules on tobacco cannabis and amphetamine use, violence, and a range of other health related behaviors, such as hygiene and physical activity, as well as demographics such as age and gender.^2^ The study variables are described in Table 1.

### Statistical analysis

Descriptive statistics were used to describe the characteristics of participants and patterns of illicit drug use. Logistic regression was used for assessing the contribution of potential predictors (sociodemographic factors such as age and gender, personal attributes such as anxiety and current smoking, parental attributes such as parental tobacco use and parental monitoring, and environmental stressors such as experiencing hunger and being bullied) on lifetime cannabis and lifetime amphetamine use. All analyses were adjusted for the multi-stage stratified cluster sampling strategy, and performed using STATA software version 12.0 (Stata Corporation, College Station, TX, USA).

## Results

The sample included 38,941 school-aged adolescents (mean age 15.4 years, SD=1.5), age range 11–18 years, from Iraq (response rate=88%), Kuwait (85%), Malaysia (89%), Mongolia (88%), and Vietnam (response rate=96%). The range of students participating per country ranged from 2,038 in Kuwait to 25,507 in Malaysia. Across all respondents from all countries, lifetime cannabis use was 0.9%, ranging from 0.6% in Vietnam to 3.2% in Kuwait, and lifetime amphetamine use was 1.0%, ranging from 0.2% in Vietnam to 3.1% in Kuwait. The prevalence of past month cannabis use was 2.1% in Iraq, 3.1% in Kuwait, 0.9% in Malaysia, and 1.1% in Mongolia (Table 2).

In multivariable unconditional regression analysis, male gender in Kuwait (OR=11.17, 95% CI=2.97, 42.02) and Mongolia (OR=2.01, CI=1.03, 3.95) was associated with lifetime cannabis use. In terms of personal attributes, anxiety (OR=2.48, 95% CI=1.19, 5.20) and suicidal ideation (OR=3.91, 95% CI=1.63, 9.35) in Kuwait, current smoking in all five countries (OR ranging from 5.25 in Mongolia to 38.12 in Vietnam), and school truancy (OR=6.70, 95% CI=2.65, 16.96) in Vietnam were positively associated with lifetime cannabis use. School truancy was negatively associated with lifetime cannabis use (OR=0.35, 95% CI=0.17, 0.71) in Iraq. Lack of peer support (OR=0.39, 95% CI=0.22, 0.68) in Malaysia and having positive peer support (OR=2.73, 95% CI=1.45, 5.15) in Mongolia were associated with lifetime cannabis use. In relation to parental attributes, parental or guardian tobacco use in Iraq (OR=2.59, 95% CI=1.04, 6.47) and Kuwait (OR=2.13, 95% CI=1.30, 3.49) were associated with lifetime cannabis use. While lack of parental or guardian bonding (OR=0.40, 95% CI=0.24, 0.68) in Malaysia was associated with lifetime cannabis use, parental or guardian bonding (OR=5.18, 95% CI=1.87, 14.37) in Vietnam and parental or guardian monitoring (OR=2.15, 95% CI=1.18, 3.90) in Malaysia were positively associated with lifetime cannabis use. With environmental stressors, food insecurity (OR=9.77, 95% CI=3.03, 31.56) in Iraq, being bullied (OR=11.26, 95% CI=1.83, 69.38) in Vietnam, and having been physically attacked (OR=2.14, 95% CI=1.02, 4.46) in Mongolia were associated with lifetime cannabis use (Table 3).

In multivariable unconditional regression analysis, male gender (OR=4.76, 95% CI=1.10, 20.55) in Kuwait, younger age (OR=0.78, 95% CI=0.63, 0.98) in Malaysia, and older age (OR=1.84, 95% CI=1.07, 3.15) in Vietnam were associated with lifetime amphetamine use. In terms of personal attributes, anxiety (OR= ranging from 2.29 in Kuwait to 3.82 in Iraq), current smoking (OR=ranging from 2.68 in Mongolia to 17.23 in Vietnam) in all countries, suicidal ideation in Kuwait (OR=3.19, 95% CI=1.35, 7.55), Malaysia (OR=2.28, 95% CI=1.26, 4.12) and Vietnam (OR=6.86, 95% CI=1.07, 43.91), and school truancy in Malaysia (OR=2.31, 95% CI=1.40, 3.81), Mongolia (OR=1.90, 95% CI=1.11, 3.28) and Vietnam (OR=9.12, 95% CI=3.06, 27.19) were associated with lifetime amphetamine use. Lack of peer support (OR=0.35, 95% CI=0.20, 0.62) was associated with lifetime amphetamine use in Malaysia. In relation to parental attributes, no associations were found with lifetime amphetamine use. With environmental stressors, experiencing food insecurity (OR=4.98, 95% CI=1.35, 18.33) in Iraq, being bullied in Iraq (OR=5.16, 95% CI=1.55, 17.18), Malaysia (OR=2.51, 95% CI=1.55, 4.08) and Vietnam (OR=9.16, 95% CI=1.05, 79.60), and having been physically attacked in Kuwait (OR=2.27, 95% CI=1.14, 4.52) and Mongolia (OR=3.07, 95% CI=1.55, 6.07) were associated with lifetime amphetamine use (Table 4).

## Discussion

In this study of school-going adolescents in five Asian countries from Middle East, Central Asia, and Southeast Asia the prevalence of lifetime cannabis and amphetamine use was found to be generally lower than previously reported in North America and Europe,^13^,^14^ India,^11^ Iran^10^ and Saudi Arabia,^12^ but was similar to previously reported results in Malaysia.^6^,^7^ Compared to the studied countries, the higher prevalence of cannabis and possibly amphetamine use in North America and Europe may be related to a greater cannabis liberalization.^13^

This study demonstrated a large geographic variation in the prevalence of lifetime and past month cannabis use and lifetime amphetamine use, with a higher prevalence in Iraq and Kuwait, and lower prevalence in Malaysia, Mongolia, and Vietnam. The higher prevalence of cannabis and amphetamine use in Iraq may be explained by a high degree of exposure to environmental stressors, such as ongoing violence and unrest, as well as the experience of hunger or food insecurity. The relatively low lifetime amphetamine use in Southeast Asia (Malaysia and Vietnam) in this study may reflect a further stabilizing of the past “peak of the methamphetamine epidemic” in Southeast Asia.^27^ The low prevalence of illicit drug use in Malaysia may also be attributed to school prevention programs.^7^

Overall, the study found that a higher number of males when compared to females were lifetime cannabis users in Kuwait and Mongolia. Lifetime amphetamine use among males in Kuwait was higher in males. There was no significant gender difference in the other countries, which is an interesting finding. The role of gender in substance abuse requires further investigation^28^, as UNODC^29^ noted that the “gender gap (i.e. the difference between the prevalence of substance use among males and females) has in fact been closing.” The gender difference in Kuwait and Mongolia may reflect stronger gender role differences that predispose men to engage in substance use behavior when compared with Iraq, Malaysia, and Vietnam.^20^

The study found that adolescents who were current smokers were more likely to be lifetime amphetamine users. This seems to indicate that certain problem behaviors may become a trend during adolescence, and the use of one drug may lower the barriers of taking another drug.^30^ Poly-drug use (tobacco use and cannabis and/or other drugs) has also been reported by previous studies^18^,^31^,^32^ and suggests the need for poly-drug use interventions.

When it comes to personal attributes, mental distress (anxiety and suicidal ideation) and school truancy were found to be associated with cannabis and amphetamine use in several countries that we investigated, corroborating previous studies in Malaysia and Africa.^7^,^19^–^21^ Having mental distress may increase adolescents’ vulnerability to drug use.^16^ Adolescents who are mentally distressed may use cannabis and/or amphetamine to alter their well-being,^33^ or they may want to cope with mental distress by using illicit drugs.^20^ Illicit drug use or school truancy may be seen as a marker of other deviant behaviors, which may lead to a greater likelihood of experimenting with cannabis and/or amphetamine use outside of school settings.^34^ In agreement with a previous study,^5^ this study found an association between lack of peer support and cannabis and amphetamine use in Malaysia, but not in the other countries. Strong peer relations or support may help to protect from illicit drug use.

Regarding parental attributes, this study corroborated a study from Ghana,^23^ suggesting that parental tobacco use was associated with lifetime cannabis use. Parents play an important role in the formation of norms and practices among adolescents.^35^ Adolescents are more likely to engage in similar behavior as their parents when it comes to substance abuse behaviors.^35^ Although parental or guardian monitoring and/or bonding was protective from cannabis use in Malaysia, none of the parental support measures were protective in relation to lifetime amphetamine use, unlike in findings from previous investigations.^7^,^21^,^24^ Parental monitoring and bonding behavior may demonstrate concern and support, which may prevent children from the development of illicit drug use habits.^23^

In agreement with previous studies,^17^–^19^,^21^ this study found that environmental stressors, including experiencing hunger (or low socioeconomic status), being bullied and having been physically attacked, were associated with lifetime cannabis and/or amphetamine use in several countries. It is possible that adolescents who experience various forms of environmental stressors are more likely to associate themselves with deviant peers in trying to cope with a stressful environment, and thus more likely engage in illicit drug use.^16^

Due to the cross-sectional study design, causal inferences cannot be made. Further, the self-report of illicit cannabis and amphetamine use may be interpreted with caution because of poor memory recall and possibly underreporting of illicit drug use. Moreover, several indicators, such as anxiety and loneliness were only measured with single question items, which has its limitations. Some of the variables (anxiety, parental tobacco use, and food insecurity) assessed in the GSHS were not available for all the countries our study was focusing on, which need to be further investigated.

This study found that relatively low proportion of adolescents in five Asian countries engage in cannabis and amphetamine use, and identified various risk factors associated with its use. Our findings contribute to the body of knowledge on illicit drug use among adolescents in Asian countries, which can facilitate effective global policy responses. School health policy and interventions should target the prevention of cannabis and amphetamine use, while taking into account environmental stressors and personal attributes such as anxiety and school truancy. Translational research may help in identifying the risk factors most amenable to address or change in the future public health interventions focusing on reduction in illicit drug use. Future prospective studies are needed to identify causal relationships among personal attributes, parental attributes, environmental stressors, and illicit substance use.

## Figures and Tables

**Table 1:** Description of variables

Variables	Question	Response options
Cannabis use	“During your life, how many times have you used marijuana (also called hashish)?” or other country specific names	1=0 times, 2=1 or 2 times, 3=3–9 times, 4=10–19 times and 5==20 or more times (coded 1=0 and 2–5=1)
	“During the past 30 days, how many times have you used marijuana (also called hashish)?” or other country specific names	1=0 times, 2=1 or 2 times, 3=3–9 times, 4=10–19 times and 5==20 or more times (coded 1=0 and 2–5=1)
Amphetamine use	“During your lifetime, how many times have you used amphetamines or methamphetamines (also called Parkizol or Artane)?” or other country specific names	1=0 times, 2=1 or 2 times, 3=3–9 times, 4=10–19 times and 5==20 or more times (coded 1=0 and 2–5=1)
**Personal attributes**		
Anxiety	“During the past 12 months, how often have you been so worried about something that you could not sleep at night?”	1=never to 5=always (coded 1–3=0 and 4–5=1)
Loneliness	“During the past 12 months, how often have you felt lonely?”	1=never to 5=always (coded 1–3=0 and 4–5=1)
Suicidal ideation	“During the past 12 months, did you ever seriously consider attempting suicide?”	1 = yes, 2 = no
Current smoking cigarettes	“During the past 30 days, on how many days did you smoke cigarettes?”	1=0 days to 7=All 30 days (coded 1=0 and 2–7=1)
School truancy	“During the past 30 days, on how many days did you miss classes or school without permission?”	1=0 days to 5= 10 or more days (coded 1=0 and 2–5=1)
Peer support	“During the past 30 days, how often were most of the students in your school kind and helpful?”	1=never to 5=always (coded 1–3=0 and 4–5=1)
**Parental attributes**		
Either or both parents use tobacco	Which of your parents or guardians use any form of tobacco?	1=neither, 2=my father or male guardian, 3=my mother or female guardian
Parental monitoring	“During the past 30 days, how often did your parents or guardians check to see if your homework was done?”	1=never to 5=always (coded 1–3=0 and 4–5=1)
	“During the past 30 days, how often did your parents or guardians go through your things without your approval?”	1=never to 5=always (coded 1–3=0 and 4–5=1)
Parental connectedness	“During the past 30 days, how often did your parents or guardians understand your problems and worries?”	1=never to 5=always (coded 1–3=0 and 4–5=1)
Parental bonding	“During the past 30 days, how often did your parents or guardians really know what you were doing with your free time?	1=never to 5=always (coded 1–3=0 and 4–5=1)
**Environmental stressors**		
Hunger	“During the past 30 days, how often did you go hungry because there was not enough food in your home?”	1 = never to 5 = always (coded 1–3=0 and 4–5=1)
Bullied	“During the past 30 days, on how many days were you bullied?”	1=0 days to 7=All 30 days (coded 1=0 and 2–7=1)
In a physical fight	“During the past 12 months, how many times were you in a physical fight?”	1=0 times to 8=12 or more times (coded 1=0 and 2–8=1)
Physically attacked	“During the past 12 months, how many times were you physically attacked?”	=0 times to 8=12 or more times (coded 1=0 and 2–8=1)

**Table 2:** Prevalence of drug use in five Asian countries

Variable	Sample size	Lifetime cannabis use			Lifetime amphetamines use			Past month cannabis use
Country (study year)		All	Males	Females	All	Males	Females	All

		N (%)	N (%)	N (%)	N (%)	N (%)	N (%)	N (%)
Iraq (2012)	2038	(2.4)	30 (3.0)	13 (1.6)	(2.6)	27 (2.7)	20 (2.4)	42 (2.2)
Kuwait (2011)	2672	(3.2)	77 (5.7)	6 (0.4)	(3.1)	61 (5.0)	8 (0.7)	80 (3.1)
Malaysia (2012)	25507	(0.9)	173 (1.5)	37 (0.4)	(1.0)	179 (1.6)	37 (0.4)	208 (0.9)
Mongolia (2013)	5393	(1.3)	43 (1.8)	22 (0.8)	(1.7)	43 (1.9)	36 (1.3)	53 (1.1)
Vietnam (2013)	3331	(0.6)	14 (6.7)	4 (1.1)	(0.2)	12 (0.9)	4 (0.3)	NA
All	38941	(0.9)	337 (1.5)	82 (0.4)	(1.0)	332 (1.4)	105 (0.6)	383 (1.5)

NA = Not assessed

**Table 3:** Factors associated with lifetime cannabis use

Variable	AOR (95% CI)	AOR (95% CI)	AOR (95% CI)	AOR (95% CI)	AOR (95% CI)
	Iraq	Kuwait	Malaysia	Mongolia	Vietnam
**Sociodemographic**					
Age (years)	0.63 (0.35, 1.11)	0.99 (0.75, 1.31)	1.00 (0.80, 1.26)	0.93 (0.74, 1.17)	1.16 (0.66, 2.02)
Gender					
Females (48.2%)	Reference	Reference	Reference	Reference	Reference
Males (51.8%)	0.75 (0.14, 3.91)	11.17 (2.97, 42.02)***	1.51 (0.77, 2.99)	2.01 (1.03, 3.95)*	0.93 (0.05, 16.98)
**Personal attributes**					
Anxiety (14.4%)	0.77 (0.29, 2.04)	2.48 (1.19, 5.20)*	1.73 (0.89, 3.36)	2.01 (0.79, 5.14)	Not assessed
Loneliness (15.3%)	2.02 (0.74, 5.50)	1.35 (0.60, 3.04)	1.91, 0.98, 3.71)	1.09 (0.45, 2.67)	1.82 (0.36, 9.23)
Suicidal ideation (19.1%)	2.52 (0.95, 7.05)	3.91 (1.63, 9.35)**	1.74 (0.87, 3.50)	1.46 (0.56, 3.83)	1.69 (0.29, 9.93)
Current smoking (8.9%)	9.20 (3.62, 23.41)***	7.30 (2.34, 22.73)**	11.06 (5.41, 22.60)***	5.26 (2.35, 11.81)***	38.12 (8.02, 181.19)***
School truancy (36.6%)	0.35 (0.17, 0.71)**	1.34 (0.84, 2.13)	2.07 (0.90, 4.76)	1.86 (0.88, 3.97)	6.70 (2.65, 16.96)***
Peer support (29.2%)	0.60 (0.13, 2.72)	0.65 (0.26, 1.62)	0.39 (0.22, 0.68)***	2.73 (1.45, 5.15)**	1.41 (0.51, 3.89)
**Parental attributes**					
Either or both parents use tobacco (21.4%)	2.59 (1.04, 6.47)*	2.13 (1.30, 3.49)**	1.03 (0.71, 1.49)	1.34 (0.65, 2.77)	Not assessed
Parental or guardian monitoring (25.6%)	0.40 (0.12, 1.32)	0.99 (0.49, 2.02)	2.15 (1.18, 3.90)*	0.69 (0.39, 1.24)	0.26 (0.04, 1.54)
Parental or guardian connectedness (31.9%)	1.13 (0.47, 2.75)	1.82 (0.50, 6.67)	1.02 (0.67, 1.57)	0.77 (0.29, 2.00)	0.40 (0.05, 3.42)
Parental or guardian bonding (36.4%)	0.22 (0.05, 1.06)	1.34 (0.66, 2.72)	0.40 (0.24, 0.68)***	0.54 (0.28, 1.04)	5.18 (1.87, 14.37)**
**Environmental stressors**					
Hunger (11.2%)	9.77 (3.03, 31.56)***	1.24 (0.48, 3.21)	1.35 (0.70, 2.61)	2.87 (0.65, 12.69)	Not assessed
Bullied (45.1%)	1.25 (0.47, 3.32)	1.70 (0.60, 4.80)	1.59 (0.96, 2.61)	0.78 (0.36, 1.66)	11.26 (1.83, 69.38)**
In physical fight (44.8%)	1.12 (0.33, 3.80)	1.01 (0.51, 2.00)	1.84 (0.84, 4.03)	1.33 (0.61, 2.86)	0.95 (0.37, 2.49)
Physically attacked (34.0%)	1.10 (0.41, 2.96)	1.43 (0.66, 3.12)	1.10 (0.64, 1.88)	2.14 (1.02, 4.46)*	0.49 (0.19, 1.26)

AOR = Adjusted Odds Ratio; CI = Confidence Interval;

*** P<0.001;

** P<0.01;

* P<0.05

**Table 4:** Factors associated with lifetime amphetamine use

Variable	AOR (95% CI)	AOR (95% CI)	AOR (95% CI)	AOR (95% CI)	AOR (95% CI)
	Iraq	Kuwait	Malaysia	Mongolia	Vietnam
**Sociodemographic**					
Age (years)	0.75 (0.51, 1.10)	0.86 (0.59, 1.26)	0.78 (0.63, 0.98)*	1.02 (0.82, 1.26)	1.84 (1.07, 3.15)*
Gender					
Females (48.2%)	Reference	Reference	Reference	Reference	Reference
Males (51.8%)	0.88 (0.20, 4.08)	4.76 (1.10, 20.55)*	1.47 (0.75, 2.90)	0.92 (0.52, 1.64)	2.78 (0.10, 79.01)
**Personal attributes**					
Anxiety (14.4%)	3.82 (1.08, 13.50)*	2.29 (1.68, 3.14)***	2.52 (1.36, 4.66)**	2.82 (1.36, 5.86)**	Not assessed
Loneliness (15.3%)	0.76 (0.13, 4.55)	1.06 (0.58, 1.94)	1.71 (0.90, 3.25)	0.91 (0.40, 2.06)	0.91 (0.16, 5.18)
Suicidal ideation (19.1%)	1.28 (0.30, 5.50)	3.19 (1.35, 7.55)*	2.28 (1.26, 4.12)**	1.32 (0.67, 2.62)	6.86 (1.07, 43.91)*
Current smoking (8.9%)	6.66 (2.03, 15.74)**	5.97 (2.92, 12.20)***	5.73 (3.47, 0.48)***	2.68 (1.28, 5.62)**	17.23 (3.07, 96.77)**
School truancy (36.6%)	0.63 (0.27, 1.49)	1.36 (0.89, 2.08)	2.31 (1.40, 3.81)***	1.90 (1.11, 3.28)*	9.12 (3.06, 27.19)***
Peer support (29.2%)	0.48 (0.16, 1.45)	0.87 (0.35, 2.13)	0.35 (0.20, 0.62)***	1.32 (0.50, 3.48)	2.27 (0.64, 7.98)
**Parental attributes**					
Either or both parents use tobacco (21.4%)	1.87 (0.76, 4.59)	1.74 (0.87, 3.49)	0.95 (0.62, 1.44)	1.35 (0.73, 2.49)	Not assessed
Parental or guardian monitoring (25.6%)	0.74 (0.25, 2.22)	0.99 (0.56, 1.78)	1.70 (0.95, 3.05)	0.62 (0.30, 1.30)	0.59 (0.05, 6.77)
Parental or guardian connectedness (31.9%)	0.40 (0.06, 2.59)	1.40 (0.51, 3.88)	0.77 (0.42, 1.40)	0.35 (0.10, 1.28)	0.13 (0.01, 3.09)
Parental or guardian bonding (36.4%)	0.45 (0.07, 2.98)	1.07 (0.52, 2.24)	0.65 (0.38, 1.14)	0.80 (0.36, 1.78)	3.62 (0.80, 16.37)
**Environmental stressors**					
Hunger (11.2%)	4.98 (1.35, 18.33)*	1.15 (0.39, 3.43)	1.35 (0.51, 3.56)	0.88 (0.10, 7.56)	Not assessed
Bullied (45.1%)	5.16 (1.55, 17.18)**	2.20 (0.63, 7.77)	2.51 (1.55, 4.08)***	1.13 (0.55, 2.34)	9.16 (1.05, 79.60)*
In physical fight (44.8%)	1.16 (0.38, 3.51)	0.97 (0.38, 2.51)	1.72 (0.99, 2.99)	2.05 (0.93, 4.53)	0.94 (0.15, 5.72)
Physically attacked (34.0)	0.62 (0.27, 1.43)	2.27 (1.14, 4.52)*	1.26 (0.77, 2.07)	3.07 (1.55, 6.07)**	0.55 (0.08, 3.56)

AOR = Adjusted Odds Ratio; CI = Confidence Interval;

*** P<0.001;

** P<0.01;

* P<0.05
